# Fentanyl Induces Cerebellar Internal Granular Cell Layer Apoptosis in Healthy Newborn Pigs

**DOI:** 10.3389/fneur.2018.00294

**Published:** 2018-05-01

**Authors:** Hemmen Sabir, John Dingley, Emma Scull-Brown, Ela Chakkarapani, Marianne Thoresen

**Affiliations:** ^1^Neonatal Neuroscience, School of Clinical Sciences, University of Bristol, St. Michael’s Hospital, Bristol, United Kingdom; ^2^Department of Pediatrics I/Neonatology, University Hospital Essen, University Duisburg-Essen, Essen, Germany; ^3^Swansea University College of Medicine, Swansea, United Kingdom; ^4^Division of Physiology, Institute of Basic Medical Sciences, University of Oslo, Oslo, Norway

**Keywords:** newborn, brain, neurotoxicity, opioids, sedation

## Abstract

**Background:**

Opioids like fentanyl are regularly used in neonates for analgesia and sedation. So far, they have been reported to be safe and eligible to use. The cerebellum has become a focus of neurodevelopmental research within the last years, as it is known to play an important role in long-lasting motor, cognitive, and other behavioral changes. The cerebellar cortex is of major importance in the coordinative role of the cerebellum and highly vulnerable to injury and impaired growth.

**Objective:**

This study was performed to evaluate the apoptotic effect of intravenous fentanyl infusion on the cerebellum in healthy newborn pigs.

**Methods:**

Thirteen healthy pigs (<median 12 h old) were randomized into (1) 24 h of intravenous fentanyl at normothermia (NTFe, *n* = 6) or (2) non-ventilated controls at normothermia (NTCTR, *n* = 7). Cerebellar sections were morphologically assessed after staining with hematoxylin–eosin. In addition, paired sections were immuno-stained for cell death [Cleaved caspase-3 and terminal deoxynucleotidyl transferase-mediated deoxyuridine-triphosphate nick-end labeling (TUNEL)], and positive cells were counted in defined areas of the internal granular cell layer. In total, cells in three cerebellar gyri were counted.

**Results:**

We found that there was an increase in cells with apoptotic morphology in the internal granular cell layer in the NTFe group. For quantification, we found a significant increase in cell death in group (1) [median (range) number of caspase-3-positive cell group (1) 8 (1–22) vs. group (2) 1 (1–6) and TUNEL-positive cells (1) 6 (1–10) vs. (2) 1 (0–4)]. In both groups, there was no difference in the number of Purkinje cells. Both groups had comparable and stable physiological parameters throughout the 24 h period.

**Conclusion:**

Twenty-four hours of continuous intravenous fentanyl infusion increased apoptosis in the internal granular cell layer in the cerebellum of healthy newborn pigs.

## Introduction

The appropriate development of the central nervous system (CNS) relies on the precise temporal–spatial pattern of complex molecular pathways guiding proliferation, migration, differentiation, and survival of neural cells (1). Interference with these pathways can disrupt physiological development patterns and may lead to permanent CNS impairments. Analgesics and sedative drugs are potent modulators of molecular pathways, and their use in preterm and term newborns has been associated with impaired long-term neurological outcomes (2). Control of pain and agitation is a fundamental component of neonatal intensive care. Opioids, especially morphine, are a commonly used analgesic in both preterm and term neonates and its use has been shown to be safe (3–5). However, high doses of morphine have also been shown to be associated with increased risk of brain injury in preterm infants (6). Fentanyl, a more potent opioid, has become an alternative to morphine in preterm and term infants. Small randomized-controlled trials have shown reduced stress responses in ventilated preterm infants receiving continuous fentanyl infusion, with no increased incidence of brain injury (7, 8). However, current clinical (2) and preclinical (9) data show that continuous fentanyl infusion may alter cerebellar development, leading to cerebellar hypoplasia and long-term impairments. In addition, perinatal opioid exposure has been shown to lead to cerebellar neuronal loss and cerebellar dysfunction (10).

The cerebellum has become a focus of neurodevelopmental research within the last years, as it is known to play an important role in long-lasting motor, cognitive and other behavioral changes (11–14). Input from the cerebellar cortex has a major role in the functioning of the cerebellum (coordination, precision, and accurate timing). The cerebellum is very vulnerable to injury and impaired growth (12). As the basic architecture of the cerebellar cortex is comparable between pigs and humans (15), we aimed to evaluate the apoptotic effect of continuous intravenous fentanyl on the cerebellum in healthy newborn pigs.

## Materials and Methods

### Conduct of Experiment

All experiments were conducted according to the United Kingdom Home Office license guidelines and were approved by the University of Bristol Ethical Review Panel (Bristol, United Kingdom). The experimental setup is detailed in the larger experiment, where we reported the safety of 50% Xenon (Xe) ventilation in healthy newborn pigs, showing that ventilation with 50% Xe does not cause cellular injury to the newborn cerebrum (16). This study uses data from 13 healthy newborn pigs (median age 10 h, interquartile range 9–12 h) receiving intravenous fentanyl sedation, while being mechanically ventilated at normothermia (*n* = 6) or serving as control animals without special treatment (*n* = 7).

### Animal Preparation, Baseline Data, and Management of Pigs

All animals were handled as previously published (16). In brief, after initial intubation, insertion of umbilical arterial and venous catheters, continuous monitoring of mean arterial blood pressure and heart rate was enabled in the fentanyl treatment group (NTFe group) and pigs were subsequently extubated and self-ventilating in air. Physiological parameters, mean arterial blood pressure and heart rate, were continuously recorded. Intensive care management was performed as previously described with 5 ml/kg/h intravenous maintenance fluid (5% dextrose/0.45% saline) in addition to being bottle fed with pig formula (Pig formula milk “Baby Lactal”; Peter Möller A/S, Oslo, Norway) at a rate of ~10 ml/kg/h. Control pigs (NTCTR group) were self-ventilating in air and bottle fed every 2–3 h with pig formula to maintain a similar fluid intake. Blood sampling was undertaken from the inserted lines at preset time points, as well as when clinically indicated. Frequent temperature measurements were undertaken with a rectal probe (reusable YSI 400 series, CritiCool, MTRE, Yavne, Israel) inserted 6 cm into the rectum, and a skin probe (CritiCool, MTRE, Yavne, Israel) sited on the ear lobe. Both probes were calibrated before use within ±0.1°C, over a temperature range of 20–40°C, against a certified mercury-in-glass thermometer (BS593; Zeal, London, United Kingdom). Rectal temperature (*T*_rec_) was maintained at 38.5 ± 0.2°C using a servo-controlled (CritiCool, MTRE, Yavne, Israel) mat containing circulating water.

### Fentanyl Sedation

After intubation and vascular umbilical cord access, continuous fentanyl infusion was started with a bolus of 10 µg/kg followed by maintenance infusion with 1 µg/kg/h. Thereafter, the fentanyl infusion was adjusted to achieve adequate sedation and tolerance of the central and continuous arterial blood pressure monitoring lines. Mean arterial blood pressure was higher than 40 mmHg in all pigs throughout the 24-h treatment period, providing an adequate cerebral blood flow for newborn pigs (16–18).

### Neuropathology Assessment

**Figure 1:**
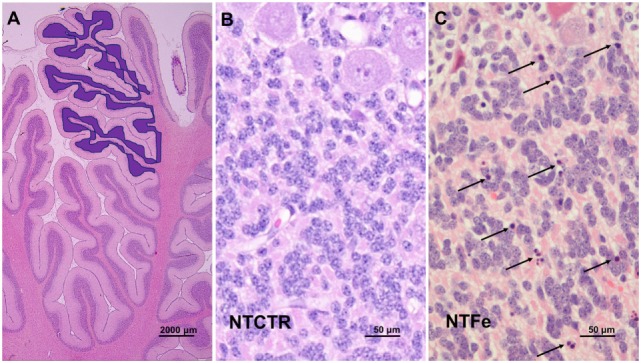
Photomicrography of histological features. **(A)** Assessed area of the internal granular cell layer of three complete gyri of the anterior cerebellar lobe as highlighted in blue. **(B,C)** Representative images of the inner granular cell layer of the NTCTR and NTFe groups are shown. Arrows indicate cells with homogenous eosinophilic cytoplasm and pyknotic nuclei.

After 24 h of allocated treatment, all pigs were intubated and deeply anesthetized with isoflurane (16). Brains were slowly flushed with 0.9% saline through the common carotid arteries followed by perfusion fixation with 10% neutral buffered formalin and dissected out. The cerebellum was removed and the hemispheres divided. The right hemisphere was coronally cut into 5-mm blocks and paraffin embedded. Two representative blocks of the left cerebellum were chosen, best presenting the cortex and white matter regions of the cerebellum (Figure 1A). Hematoxylin and eosin (H&E)-stained 5-µm thick sections were assessed at 40× magnification. Three complete gyri of the anterior lobe of the cerebellum were assessed (Figure 1A), and cells were scored as apoptotic when showing typical morphology of apoptosis (19).

### Immunohistochemistry

Immunohistochemical staining was performed as previously described (16). Briefly, slides were prepared from paraffin-embedded sections. For quantification of apoptotic cells, two adjacent sections were stained with Cleaved caspase-3. Primary rabbit antibody against Cleaved caspase-3 [1:500, polyclonal rabbit anti-Cleaved caspase-3 (ASP175) Cell Signalling Technologies] was applied overnight at room temperature. In addition, for the assessment of DNA fragmentation, the adjacent sections were stained with terminal deoxynucleotidyl transferase-mediated deoxyuridine-triphosphate nick-end labeling (TUNEL). TUNEL staining was performed as instructed by the manufacturer (TUNEL AP, cat. no. 11772457001, Roche).

For each animal, three complete gyri were counted for Cleaved caspase-3 and TUNEL-positive cells at 40× magnification. Total cell counting was performed in three non-overlapping fields, each sized 2,000 µm × 200 µm representing the three gyri assessed by H&E staining, by three independent observers blinded to the randomization and to clinical details of the pigs.

### Statistical Analysis

Statistical analysis was performed with SPSS version 22 (SPSS Inc., Chicago, IL, USA). The Wilcoxon test was used for the two-group comparison. To assess the effect of sex and age since birth in hours on the number of Cleaved caspase-3 and TUNEL-positive cells, regression analysis was used. Two-sided testing with *p* < 0.05 was considered statistically significant. Data are presented as median (interquartile range).

## Results

### Physiological Data

There were no significant differences in baseline physiological parameters between the NTFe and the NTCTR group (Table 1). Blood gases, blood glucose, and lactate values were within the normal range in all animals.

**Table 1:** Baseline parameters during 24 h treatment period.

	NTFe (self-ventilating, 24 h iv fentanyl at normothermia, *n* = 6)	NTCTR (self-ventilating, controls at normothermia, *n* = 7)
Median weight [kg, (IQR)]	1.75 (1.63–1.86)	1.36 (1.29–1.42)
Sex	2 females, 4 males	4 females, 3 males
Median age [h, (IQR)]	10 (9–12)	10 (9.5–11)
Median heart rate [/min, (IQR)]	159 (153–164)	n/a
Median arterial blood pressure [mmHg, (IQR)]	53 (46.8–55)	n/a
Median tcSO_2_ [%, (IQR)]	98 (97–99)	n/a
Median pH (IQR)	7.41 (7.38–7.45)	7.46 (7.42–7.50)
Median glucose [mmol/l, (IQR)]	5.9 (5.4–6.1)	3.9 (3.4–4.3)
Median lactate [mmol/l, (IQR)]	2.1 (1.4–3.1)	3.3 (2.9–3.5)
Median fentanyl dosage [μg/kg/h, (IQR)]	17.5 (15–20)	0

*Median (interquartile range) baseline parameters (weight, sex, and age), heart rate, arterial blood pressure, transcutaneous oxygen saturation (tcSO_2_), pH, blood glucose, and lactate levels of all 13 animals in the two treatment groups and median fentanyl dosage for 6 pigs receiving iv fentanyl*.

*IQR, interquartile range*.

*n/a: Data were not applicable; these animals were not cardiovascularly monitored as they were non-instrumented control animals (NTCTR)*.

### Histological Results

There was a notable difference in H&E-stained sections of the internal granular cell layer in the NTFe group compared with the NTCTR group (Figures 1B,C). Cells in the NTFe group appeared with nuclear condensation and fragmentation as seen when thought to undergo apoptotic cell death (Figure 1C). As previously described, the Purkinje cell layer showed no signs of apoptosis.

### Immunohistochemistry

**Figure 2:**
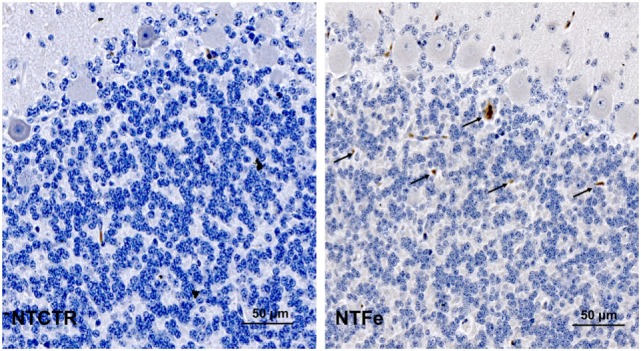
Representative images of the inner granular cell layer after Cleaved caspase-3 staining of the NTCTR and NTFe groups. Arrows indicate Cleaved caspase-3-positive cells.

**Figure 3:**
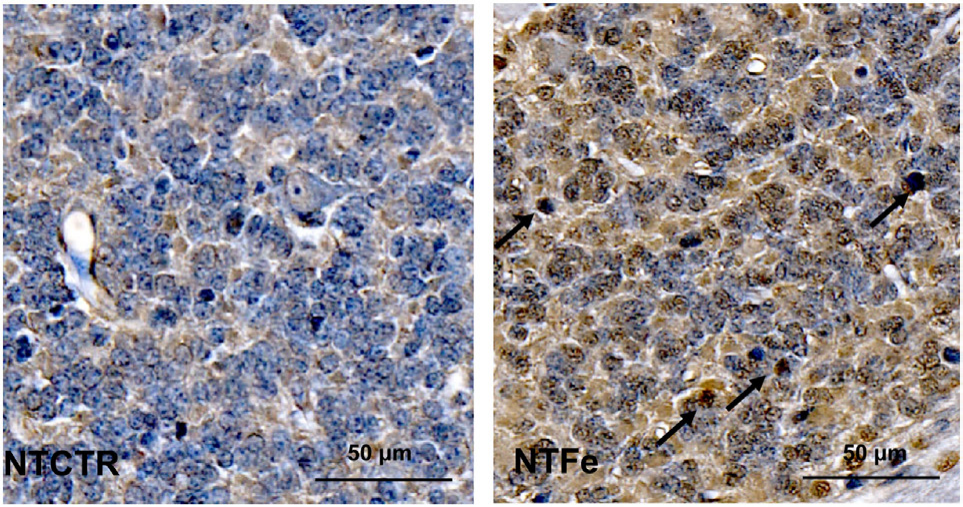
Representative images of the inner granular cell layer after terminal deoxynucleotidyl transferase-mediated deoxyuridine-triphosphate nick-end labeling (TUNEL) staining of the NTCTR and NTFe groups. Arrows indicate TUNEL-positive cells.

Immunohistochemistry showed a significant increase of Cleaved caspase-3 (*p* = 0.035) and TUNEL (*p* = 0.023) positive cells in the internal granular cell layer of pigs from the NTFe group compared with the NTCTR group, analyzed in the standardized area of tissue (Tables 2 and 3; Figures 2 and 3). Regression analysis showed no effect of sex or age on Cleaved caspase-3 or TUNEL-positive cells.

**Table 2:** Cleaved caspase-3-positive cell counting results.

	*N*	Purkinje cells	Inner granular cell layer
NTFe	6	0	8 (1.78–16.21)
NTCTR	7	0	1 (0.23–3.76)

*Total cell count for Cleaved caspase-3-positive cells per standardized area of tissue. Results are presented as median (interquartile range) number of Cleaved caspase-3-positive cells per three non-overlapping fields, each sized 2,000 µm × 200 µm. In the analyzed area, there was a significant increase in Cleaved caspase-3-positive cells in the NTFe group in the inner granular cell layer in the two-group comparison*.

*NTFe: 24 h iv fentanyl at normothermia*.

*NTCTR: controls at normothermia*.

**Table 3:** Terminal deoxynucleotidyl transferase-mediated deoxyuridine-triphosphate nick-end labeling (TUNEL) cell counting results.

	*N*	Purkinje cells	Inner granular cell layer
NTFe	6	0	6 (1.3–8.69)
NTCTR	7	0	0 (0.45–2.45)

*Total cell count for TUNEL-positive cells per standardized area of tissue. Results are presented as median (interquartile range) number of TUNEL-positive cells per three non-overlapping fields, each sized 2,000 µm × 200 µm. In the analyzed area, there was a significant increase in TUNEL-positive cells in the NTFe group in the inner granular cell layer in the two-group comparison*.

*NTFe: 24 h iv fentanyl at normothermia*.

*NTCTR: controls at normothermia*.

## Discussion

This study shows that 24 h of a clinical dose of continuous intravenous fentanyl administration significantly increases apoptotic cell death in the internal granular cell layer of the cerebellum in healthy newborn pigs. As previously reported, there was no increase of apoptosis, neither in the Purkinje cell layer in the cerebellum nor in other parts of the cerebrum in the same pigs (16). The main purpose of our previous paper (16) was to investigate whether, 50% inhaled Xe gas induces apoptosis in the healthy newborn pig brain—which it did not. Our clinical feasibility study of therapeutic hypothermia (TH) + Xe in asphyxiated term newborns therefore followed this (20, 21).

Preterm and term newborns undergo various painful procedures during their stay in the neonatal intensive care unit. In particular, term asphyxiated newborns, undergoing TH, often require unavoidable painful or stressful procedures such as intubation, mechanical ventilation, or catheterization and of course a reduced core temperature of 33.5°C. It has been shown that stress reduces the neuroprotective effect of TH (22), and therefore routine sedation is required during hypothermia treatment. Opioids have long been used for neonates undergoing painful procedures. Morphine, as the most commonly used opioid in neonates, has been shown to be safe in preterm (3, 5) and term asphyxiated neonates (4) in normal clinical dosages, without causing side effects like hypotension. In the Neurological Outcome and Preemptive Analgesics in Neonates trial, continuous morphine infusion did not increase vulnerability of ventilated preterm or term neonates to adverse neurological events and no relationship among morphine use, blood pressure variability, and intraventricular hemorrhage could be determined (6). However, additional doses of morphine were associated with an increased risk of brain injury (6). It has been shown robustly in different animal models that intrauterine and postnatal morphine exposure leads to altered brain function and reduced brain growth (23, 24). It might be that in children, standard outcome measures at 2 years of age, do not fully answer the question of long-term safety. Another explanation might be that the newborns in need of continuous opioid infusion are the sickest of preterm and term children, with many other risk factors for impaired neurological long-term outcome. Opioid analgesics act on different opioid receptors (μ-, δ-, or κ-type), which after activation, initiate multiple intracellular signaling cascades (25, 26). Of concern, these signaling pathways are implicated in various other biological processes, including the modulation of proliferation, survival, and differentiation of the neural stem cells, neurons, or glia cells (25, 27). These modulations might alter brain development, and therefore further detailed analysis of the developing brain is needed.

The cerebellum has become a focus of neurodevelopmental research within the last few years, as it is known to play an important role in long-lasting motor, cognitive, and other behavioral changes (11–14). The cerebellar cortex is of major importance to the main roles of the cerebellum (coordination, precision, and accurate timing) and highly vulnerable to injury and impaired growth (12). The basic architecture of the cerebellar cortex is comparable between pigs and humans (15), consisting of the internal granular cell layer, the Purkinje cell layer, and the superficial molecular layer (28). During the third trimester, a rapid cerebellar growth takes place (29). During normal development, the Purkinje neurons are the first neurons to be generated, and they are already mature during early fetal life (12). These Purkinje neurons are important, as they are the only efferent cells, projecting to the outside of the cerebellar cortex (30). The internal granular cell layer forms an important filter of information between mossy fiber inputs and the Purkinje cells (31). Around the time of birth and during postnatal life, the internal granular cell layer is highly active, as granular cells from the external granular cell layer migrate radially inward along the Bergmann glia to the internal granular cell layer (12, 30). During this migration phase, the internal granular cell layer is highly vulnerable. This has also been described in newborn pigs (32). Comparable to humans, the pig cerebellum is not fully mature at birth (32), and full maturation appears several months after birth (33, 34). However, the external granular cell layer is not visible in term born newborn pigs making the brain slightly more mature compared with human newborns. Whereas the granular cells play an important filter between incoming information *via* Mossy fibers and outgoing information *via* Purkinje cell axons, altered growth, and development will have long-lasting effects on cerebellar function. Strackx et al. have previously shown in a fetal sheep model that prenatal intra-amniotic injection of lipopolysaccharide, mimicking chorioamnionitis, leads to altered granule cells and astrocytes in the internal granular cell layer, without affecting Purkinje cells or cell layer volumes (35). Even though they found an increase in granule cells, they also showed that the Purkinje cells were not altered by intra-amniotic infection. As in the newborn pig, the Purkinje neurons in the fetal sheep are already present early during fetal development, not being vulnerable around the time of birth. This explains our finding of normal Purkinje cell counts in our experimental setup. However, in this study, we demonstrate acute apoptosis in the internal granular cell layer, most likely caused by the continuous fentanyl administration. Due to the present acute experimental setup and animal legislation, we are unable to undertake long-term survival studies showing possible long-lasting effects like cerebellar growth impairment and altered neuro-functional outcome. However, it has been shown in neonatal rodents that intrauterine (10) and postnatal morphine exposure alters cerebellar growth and Purkinje cell survival (9, 36, 37). Compared with large animal models (pigs or sheep), the rodent cerebellum develops and matures postnatally, and therefore Purkinje cells are highly vulnerable in rodents, explaining the mentioned results. The use of fentanyl in preterm and term infants has increased in the last years, even though little is known regarding its effect on brain development and maturation (38, 39). Fentanyl is a potent synthetic μ-opioid receptor agonist. Small randomized-controlled trials claimed to have shown its feasibility and safety during continuous infusion in preterm infants (7, 8). We show here that fentanyl increases apoptosis in the internal granular cell layer of healthy newborn pigs. In preterm infants, McPherson et al. have shown that high cumulative fentanyl doses in preterm infants correlate with a higher incidence of cerebellar injury and lower cerebellar diameter at term equivalent age assessed by magnetic resonance imaging (MRI) (2). Both studies raise concerns over cumulative fentanyl use in preterm and term neonates. In addition, Zwicker et al. showed that preterm infants exposed to high cumulative morphine exposures had impaired cerebellar growth in the neonatal period and poorer neurodevelopmental outcomes in early childhood (40). As the cerebellum has not been the focus of previous reports on the safety and outcome of morphine or fentanyl use in preterm and term neonates, ours and the before mentioned results raise new concerns regarding its use in this patient population. Due to enhanced MRI imaging techniques, the focus of researchers and clinicians on the developing cerebellum is growing. Disruption of normal cerebellar development due to cell death in the internal granular cell layer may have long-lasting neurobehavioral effects.

There are limitations to our study. First, median, and therefore cumulative fentanyl dosages were within the higher range of normal dosage in pigs. However, we used these high dosages, as healthy pigs were self-ventilated in addition to the set mechanical ventilatory rates under fentanyl sedation, requiring high dosages of fentanyl in the original paper (16). Even though our dosages were much higher than the ones normally used in neonates, we did not experience side effects like arterial hypotension or apnea. Therefore, we claim that the increased apoptosis is due to the cumulative fentanyl dose. Second, we did not analyze long-term outcome in our study, due to the acute experimental setup and animal legislation of the original study. From the findings in fetal sheep (35) or rodents (9, 10, 36), one would expect to find long-term deficits and cerebellar growth restriction in our pigs as compared with the other animal models. Third, we only performed a subgroup analysis with a limited number of animals. However, as our data robustly shows a significant increase of apoptosis in pigs exposed to high intravenous fentanyl exposure, we do not believe that enlarged group sizes would have shown different results. Last, further detailed caspase-3-dependent apoptotic pathway analyses would have strengthened our findings and might have led to the investigation of mechanisms of fentanyl induced apoptosis in our pigs. However, we were unable to perform these analyses at the current time point due to the retrospective character of the study.

Control of pain and agitation is a fundamental component of neonatal intensive care. TH is certainly stressful, even though being the only available standard treatment for neonatal encephalopathy (41). TH reduces the risk for death and adverse neurodevelopmental outcome in moderately asphyxiated newborns (42). There is increasing use of TH in mildly asphyxiated newborns (43). In these patients, high sedative and analgesic dosages of opioids will be needed to tolerate the stress of being cold compared with comatose patients. As these newborns will most likely not develop brain injury due to mild asphyxia, they are at high risk of cerebellar impairment due to the required use of fentanyl or morphine during TH. Careful patient selection and classification is needed to identify asphyxiated newborns developing moderate to severe encephalopathy and to prevent non-beneficial over-treatment of patients. In this study, we found that 24 h of intravenous fentanyl increased apoptosis in the internal granular cell layer in the cerebellum of healthy newborn pigs.

## Ethics Statement

All experiments were conducted according to the United Kingdom Home Office license guidelines and were approved by the University of Bristol Ethical Review Panel (Bristol, United Kingdom).

## Author Contributions

HS, JD, and MT have planned and designed the study; HS, ES-B, and JD have performed the animal experiments; HS and MT have analyzed the data; HS, JD, EC, and MT have written and corrected the manuscript.

## Conflict of Interest Statement

The authors declare that the research was conducted in the absence of any commercial or financial relationships that could be construed as a potential conflict of interest.
